# Comparison of Different Locking Mechanisms in Total Hip Arthroplasty: Relative Motion between Cup and Inlay

**DOI:** 10.3390/ma13061392

**Published:** 2020-03-19

**Authors:** Sebastian Jaeger, Maximilian Uhler, Stefan Schroeder, Nicholas A. Beckmann, Steffen Braun

**Affiliations:** 1 Laboratory of Biomechanics and Implant Research, Clinic for Orthopedics and Trauma Surgery, Heidelberg University Hospital, Heidelberg University, 69118 Heidelberg, Germany; stefan.schroeder@med.uni-heidelberg.de (S.S.); Steffen.Braun@med.uni-heidelberg.de (S.B.); 2 Clinic for Orthopedics and Trauma Surgery, Heidelberg University Hospital, Heidelberg University, 69118 Heidelberg, Germany; nick.beckmann@gmail.com; 3 Department of Orthopaedic Surgery and Traumatology, Inselspital, Bern University Hospital, 3010 Bern, Switzerland

**Keywords:** cup-inlay stability, total hip arthroplasty, disassembly forces, relative motion

## Abstract

The resulting inflammatory reaction to polyethylene (PE) wear debris, which may result in osteolysis, is still considered to be a main reason for aseptic loosening. In addition to the primary wear in hip joint replacements caused by head-insert articulation, relative motions between the PE liner and the metal cup may cause additional wear. In order to limit this motion, various locking mechanisms were used. We investigated three different locking mechanisms (Aesculap, DePuy, and Zimmer Biomet) to address the resulting relative motion between the acetabular cup and PE liner and the maximum disassembly force. A standardized setting with increasing load levels was used in combination with optically based three-dimensional measurements. In addition the maximum disassembly forces were evaluated according to the ASTM F1820-13 standard. Our data showed significant differences between the groups, with a maximum relative motion at the maximum load level (3.5 kN) of 86.5 ± 32.7 µm. The maximum axial disassembly force was 473.8 ± 94.6 N. The in vitro study showed that various locking mechanisms may influence cup-inlay stability.

## 1. Introduction

Total hip arthroplasty (THA) is one of the most successful orthopedic procedures in joint replacement [1,2]. The THA shows survival rates between 82% and 96% after 10 years, depending on the age of the patient and their physical functions [3]. However, the resulting inflammatory reaction to polyethylene (PE) wear debris, which may result in osteolysis [4], is still considered to be a main reason for aseptic loosening [5,6,7]. Therefore, wear debris production is thought to be the main factor limiting long-term survival of THA. It is known that not only can primary wear processes at the articulation sliding surfaces of hip cup and femoral head play an essential role, but in addition, secondary PE wear also takes also place at the backside of the PE liners (backside wear) due to relative motion between cup and insert [8,9,10,11]. A non-conforming fit between cup and PE liner, in combination with increased relative motion, could lead to burnishing, gouging, scratching or third-body wear on the PE backside surface [12]. In order to limit the motion between the metal shell and the liner, various locking mechanisms are used. It is assumed that a firm connection of the components will reduce the relative motion and the particle migration inside the cup system [13,14]. Another rather rare reason for revision is the dislocation between cup and PE liner, which presumably can be attributed to the acetabular locking mechanism.

The aim of this study was to evaluate specific locking mechanisms regarding the resulting relative motion between the acetabular cup and the PE liner. In addition, the maximum dissembling forces were investigated. For this purpose, three implant designs were tested, to determine if different types of locking mechanisms affect the magnitude of relative motion and dissembling force.

## 2. Materials and Methods

### 2.1. Acetabular Hip Cup Systems

**Figure 1:**
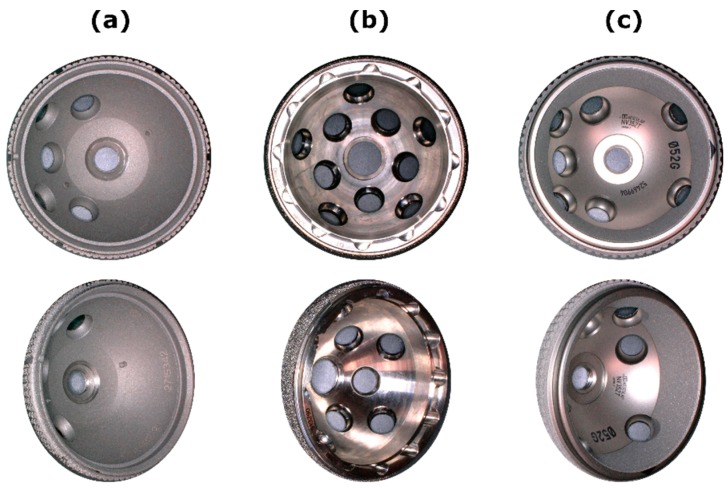
Acetabular titanium hip cup components. (**a**) Zimmer Biomet, (**b**) DePuy Synthes, (**c**) Aesculap.

Three acetabular titanium hip cup designs, with different inner designs to fixate the PE liner, were investigated (Figure 1): (A) Allofit^®^-S Alloclassic^®^ with a cross-linked Durasul^®^-PE liner (Zimmer Biomet, Warsaw, IN, USA), (B) Pinnacle^®^-Multihole combinates with a cross-linked Marathon^®^-PE liner (DePuy Synthes, Warsaw, IN, USA), (C) Plasmafit^®^ Plus7 with a conventional ultra-high-molecular-weight polyethylene (UHMWPE) liner (Aesculap, Tuttlingen, BW, Germany). All hip cup systems were non-cemented with an outer cup diameter of 52 mm and a corresponding 32 mm PE liner. In each group, six acetabular hip cups with corresponding PE liners were used.

The PE liner of the hip cup system Allofit^®^-S Alloclassic^®^ is fixed by a circular press fit mechanism. In addition, two spikes in the inner polar region of the cup on which the PE liner is inserted improve rotational stability. The inner anchoring surface of the cup is spherical, and has a smooth surface (Ra = 1 µm; Rz = 6–8 µm) [15].

The anchoring of the PE liner of the hip cup system Pinnacle^®^-Multihole is realised by a central dome region and a taper lock mechanism. Additionally, 12 grooves are placed at the rim of the shell. Corresponding to the grooves, six tabs on the liner were found to improve rotational stability. The inner anchoring surface of the cup showed values of Ra = 0.2 µm and Rz = 1 µm.

The PE liner of the hip cup system Plasmafit^®^ Plus7 is fixed by a conical locking mechanism. The cup shows a rough inner surface (Ra = 4 µm, Rz = 25 µm) [9,15].

**Figure 2:**
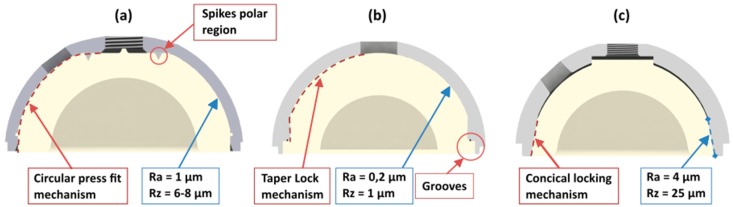
Locking mechanisms and surface specifications of the different cup systems. (**a**) Zimmer Biomet, (**b**) DePuy Synthes, (**c**) Aesculap.

An overview of the locking mechanisms and the surface specifications of the different cup systems are shown in Figure 2.

### 2.2. Test Setup

**Figure 3:**
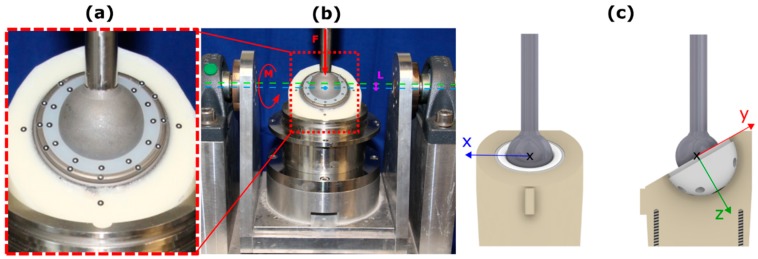
(**a**) Arrangement of the optical marker; (**b**) Relative motion measurement setup; (**c**) Camera coordinate system.

In order to carry out a standardized PE liner stability test, the cups were fixed according to Braun et al. in polyurethane (RenCast FC 53 A/B, Gößl & Pfaff GmbH, Karlskron, Germany) at an angle of 30 degrees to the vertical load axis [9]. A loading scenario was implemented using a uniaxial servo hydraulic testing machine (Bosch Rexroth, Lohr am Main, Germany). The components were loaded with an increasing dynamic load at a frequency of 1 Hz and a sinus shaped wave form. The initial load level started at 0.5 kN and increased incrementally with each load level by 1 kN, up to the maximum load of 3.5 kN. The cyclic load at each load level was maintained over 1000 cycles. The fixed acetabular cups were integrated into the testing machine and mounted on a rocker (Figure 3b). The rocker was placed on a free linear bearing to prevent transverse forces [9,15].

A rigid connection was generated between femoral head and PE liner. This, in combination with a 2 mm offset (Figure 3b L) between the centre of the implant and the rotational axis (Figure 3b green) of the rocker, initiated torque in the cup-system (Figure 3b) [9,15]. In combination with the axial compression a theoretically torque of 1, 3, 5 and 7 Nm were applied.

### 2.3. Determination of Relative Motion

The determination of relative motion was performed by placing optical markers (uncoded passive white markers, diameter 0.8 mm, GOM Item Number: 21874; GOM Gmbh, Braunschweig, Germany) along the rim of the hip cup and PE liner (Figure 3a). These markers were detected using a stereo camera system and a triangulation algorithm to calculate the 3D marker position (x-, y-, and z-axes). Based on the measured marker motion in the respective spatial direction (x, y and z direction), the resulting vector was calculated using the equation $\mathrm{XYZ}=\surd \left(X^{2}+Y^{2}+Z^{2}\right)$. In a next step, the cup and PE liner markers were separated and the resultant relative motion XYZ between both components was calculated. This calculation was carried out for every measurement time point. In a further step, the maximum of the resulting relative motion (XYZ) was determined for each individual load cycle in order to calculate the mean value of the maximum resulting relative motion for each load level (0.5, 1.5, 2.5 and 3.5 kN) [16]. The coordinate system was defined as shown in Figure 3c. The relative motion between the components was measured at cycles 10, 100, 300, 500, 800 and 995 for each load level.

### 2.4. Disassembly Test

**Figure 4:**
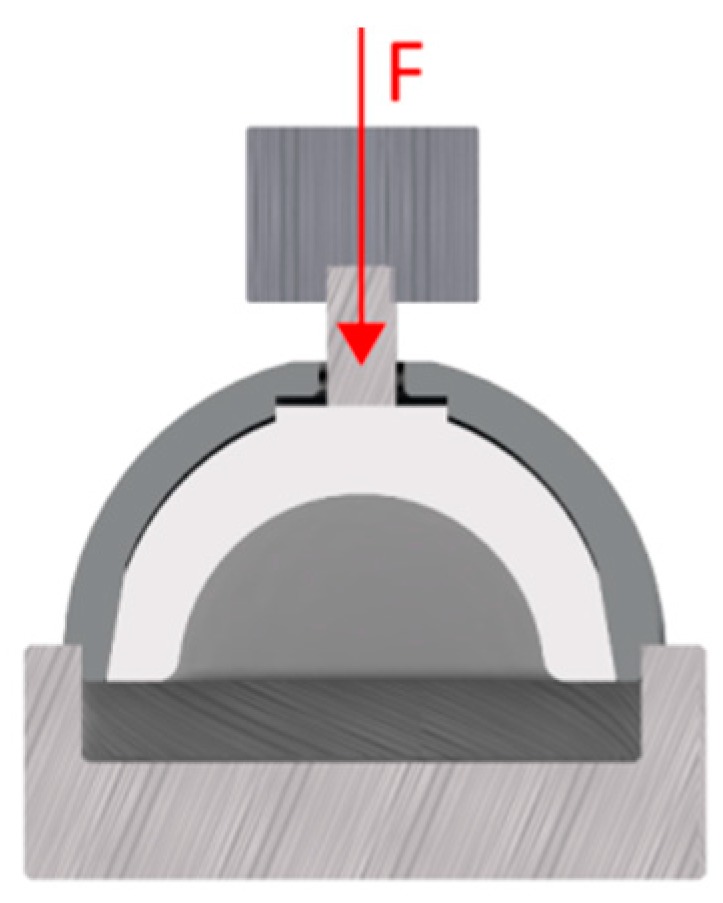
Test Setup Disassembly Test.

In addition, the maximum disassembly force of the PE Liner was determined according to the ASTM F1820-13 standard. The PE liners of the investigated cup designs were assembled in the shell with a mean peak load of 2002.2 N ± 1.1 N using a material testing machine (Zwick Roell, Z005, Ulm, Germany). Therefore, a displacement control rate of 0.04 mm/s was used. The force was applied by a femoral head coincident with the polar axis of the liner. The assembled liner shell constructs were placed in a fixture frame, with continuous support of the cup as shown in Figure 4. The axial force was applied through the centre hole of the cup with a 6 mm rod and a rate of 51 mm/min. The maximum disassembly force was recorded [17].

### 2.5. Statistical Analysis

A repeated measures analysis of variance (ANOVA) was conducted to test for significant differences in relative motion depending on the load level for all investigated cup designs. Additionally, we conducted a one-way ANOVA to assess the effect of cup design on the maximum pull out force and relative motion. To identify which particular differences between pairs of means were significant, a post hoc analysis was performed. We used a Bonferroni Test as post hoc analysis to explore differences between the three group means while controlling the experiment wise error rate. Pre-analysis, the normal distribution of the data was evaluated using a Shapiro–Wilk-test and the homogeneity of variance was verified using the Levene-test. The results allowed for the use of the ANOVA test. For the repeated measures ANOVA, a Greenhouse–Geisser adjustment was used to correct for violations of sphericity.

Additionally, the data were evaluated descriptively using the arithmetic mean, standard deviation, minimum and maximum. The data were analysed using SPSS 25 (IBM, Armonk, NY, USA) with a significance level of *p* < 0.05.

## 3. Results

### 3.1. Relative Motion

**Figure 5:**
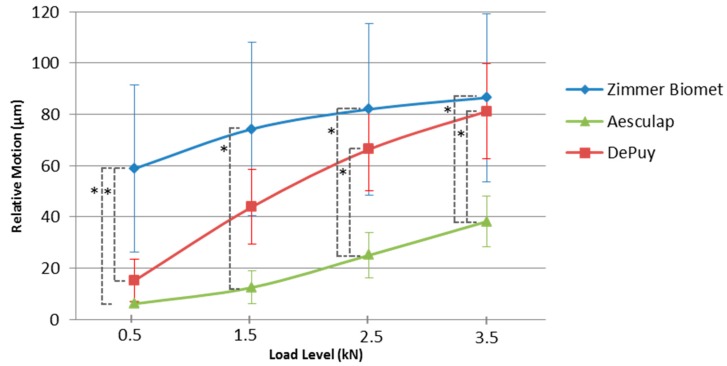
Resulting maximum relative motion, * showed significant difference.

The mean resulting maximum relative motion in XYZ-direction for each load level and all investigated cup designs are shown in Table 1 and Figure 5.

For the load level of 0.5 kN the Bonferroni-adjusted post-hoc analysis revealed a significant difference (*p* = 0.001) for mean resulting maximum relative motion of the Zimmer Biomet and the Aesculap group (52.67, 95%-CI [82.85, 22.49]) and also between the Zimmer Biomet and DePuy group (*p* = 0.004) (43.61, 95%-CI [13.43, 73.79]). There was no significant difference (*p* = 1.0) for the relative motion between the Aesculap and DePuy groups. The load level of 1.5 kN showed a significant difference (*p* < 0.001) for the relative motion between the Zimmer Biomet and the Aesculap group (61.75, 95%-CI [28.31, 95.18]). There was no significant difference between the Zimmer Biomet and DePuy groups (*p* = 0.082) or between the Aesculap and DePuy groups (*p* = 0.070). The load level of 2.5 kN showed a significant difference (*p* = 0.001) between the Zimmer Biomet and Aesculap groups (56.85, 95%-CI [22.52, 91.18]) and between the Aesculap and DePuy groups (*p* = 0.017) (41.23, 95%-CI [6.90, 75.56]). There was no statistically significant difference for the relative motion between the Zimmer Biomet and DePuy groups (*p* = 0.718). For the load level of 3.5 kN, the Bonferroni-adjusted post-hoc analysis revealed a significant difference (*p* = 0.006) for the mean resulting maximum relative motion between the Zimmer Biomet and Aesculap groups (48.28, 95%-CI [13.41, 83.14]) and between the Aesculap and DePuy groups (*p* = 0.014) (42.92, 95%-CI [8.06, 77.79]). There was no statistically significant difference for the relative motion between the Zimmer Biomet and DePuy groups (*p* = 1.0). The repeated measures ANOVA with a Greenhouse–Geisser correction determined that the load levels showed a significant influence on the relative motion, *F* (1.264, 18.964) = 309.886, *p* < 0.001.

### 3.2. Disassembly Test

**Figure 6:**
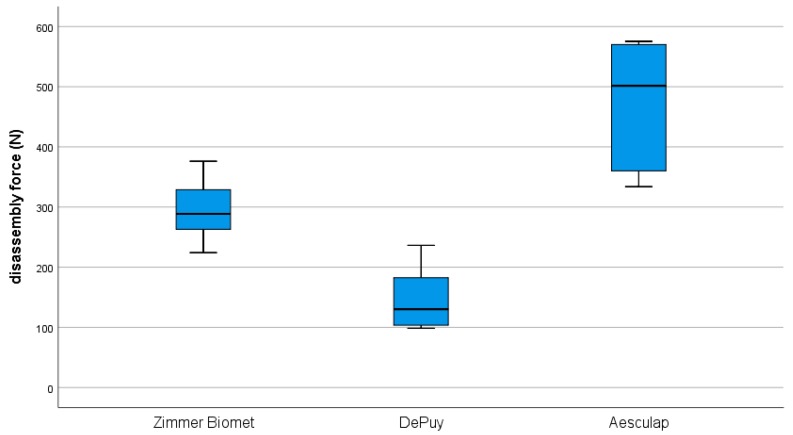
Resulting maximum axial disassembling forces.

The mean maximum axial disassembling forces showed the highest values for the Aesculap group at 473.7 ± 94.6 N, followed by the Zimmer Biomet group at 294.8 ± 48.2 N and the DePuy group at 146.8 ± 49.8 N (Figure 6). The mean maximum force differed statistically significantly for the investigated cup designs, *F* (2, 15) = 29.25, *p* < 0.001.

The Bonferroni-adjusted post-hoc analysis revealed a significant difference for the mean maximum disassembling force between the Aesculap and Zimmer Biomet groups (*p* = 0.002), (178.90, 95%-CI [63.60, 294.20]) and between the Aesculap and DePuy groups (*p* < 0.001), (326.90, 95%-CI [211.60, 442.20]). There was also a significant difference between the Zimmer Biomet and DePuy groups (*p* = 0.011), (148.00, 95%-CI [32.70, 263.30]).

## 4. Discussion

Osteolysis-induced bone destruction and the following aseptic loosening is one of the major complications of prosthetic hip replacement [5,6,7]. PE wear particles can cause osteolysis due to an inflammatory reaction. Existing publications proved that, additionally to the PE wear due to the main articulation, wear could be a consequence of the backside wear of the convex side of the PE liner [18]. The design and material could have an impact on the damage of the backside of polyethylene in modern modular acetabular cups [19]. The motion between the PE liner and the cup of a modular acetabular component exacerbated the polyethylene wear as well. This kind of micromotion, respectively, relative motion between cup and liner, is influenced by several factors. Factors could include a reduced conformity, screw holes, and different locking mechanisms and geometries [12,14,20,21].

Kurtz et al. determined the relative motion between cup and liner using a three-dimensional finite element model [12]. The focus was on considering the influence of nonconforming between liner and metal cup. Furthermore, the effects of rim and equatorial restraints were considered. The result of the study stated that backside nonconformity and locking restraints have a relevant influence on backside relative motion. Systems with a conforming cup exhibited an axial motion between 8.5 and 12.8 µm [12]. Nonconforming models offered up to 63% higher motion [12]. An in vitro study was performed by Williams et al. to determine the fixation of PE liners in metal cups [14]. Therefore, the rotational and axial motion was measured using linearly variable differential transducers. Axial loads from 0.272 kN to 2.720 kN and torsional loads from ± 7.5 Nm over 10 million cycles were applied. In the evaluation of the results, it could be derived that the rim, rotational and dome micromotion decreased as the cycles increased. Additionally an influence of the different locking mechanisms was identified. Williams et al. achieved micromotions between 3.35 ± 2.50 µm and 164.7 ± 112.5 µm after 1 million cycles [14].

Our data also showed considerable differences between cup and PE liner. Regardless of the implant design and loading situation, the range was between 6.2 ± 1.1 µm and 86.5 ± 32.7 µm. The Zimmer Biomet hip cup system with a circular snap-fit mechanism, in combination with two spikes in the inner polar region of the cup, showed relative motion between 58.8 ± 32.6 µm and 86.5 ± 32.7 µm (0.5 and 3.5 kN). The DePuy system with a central dome region and a taper lock mechanism plus grooves at the rim of the shell showed relative motion between 15.2 ± 8.3 µm and 81.1 ± 18.5 µm (0.5 and 3.5 kN). For the Aesculap system with a conical locking mechanism and a locking free contact with the base of the cup we found relative motion between 6.2 ± 1.1 µm and 38.2 ± 9.9 µm (0.5 and 3.5 kN).

For the initial load level of 0.5 kN, the DePuy and the Aesculap cups showed no significant differences (*p* = 1.0). The Zimmer Biomet cup showed significantly higher values compared to the Aesculap and DePuy cups (*p* = 0.001, *p* = 0.004). At the highest load level of 3.5 kN, the Zimmer Biomet group and DePuy system no longer showed any significant difference (*p* = 1.0). However, the Aesculap system still showed significantly lower values compared to the Zimmer Biomet and DePuy cups (*p* = 0.006, *p* = 0.014). The locking mechanism of the Aesculap system showed the lowest relative motion between cup and PE liner for the investigated load levels compared to the Zimmer Biomet and DePuy cup. A retrieval study conducted by Wasielewski et al. evaluated the backside of 55 polyethylene liners [22]. At this juncture, a distinction was made between micromotion caused by rotational instability of the insert in the shell and micromotion induced through elastic or plastic deformation. A total of 52 of the 55 inserts showed polyethylene wear on the backside in varying degrees. The most severe wear occurred in the posterior-superior and anterior-superior areas. In addition, five of six patients with acetabular osteolysis showed a high grade of backside wear, which was mainly attributed to elastic or plastic deformation related micromotion [22].

Another aspect besides the relative motion between cup and PE liner was the maximum disassembly force with regard to the risk of dislocation. Therefore, the maximum disassembly force was determined according to ASTM F1820-13 standard [17]. The maximum disassembling force showed the highest values for the Aesculap group with 473.7 ± 94.6 N, followed by the Zimmer Biomet with 294.8 ± 48.2 N and DePuy with 146.8 ± 49.8 N. In comparison, the Aesculap group showed significantly higher forces compared to the Zimmer Biomet and DePuy groups (*p* = 0.002, *p* = 0.001). There was also a significant difference between the Zimmer Biomet and DePuy groups (*p* = 0.011). In a comparative study of eight different cup systems, Tradonsky et al. determined a disassembly force between 129 N and 2950 N [23]. Our data are in the lower third compared to Tradosky et al. However, acetabular liner dissociation is a rare complication following [24].

The results of our investigation showed that a tapered locking mechanism and non-locking contact with the bottom of the cup (Aesculap) showed a significant improvement in relative motion and disassembly force compared to a system with a central dome region and a taper lock mechanism (smooth surface) plus grooves at the rim (DePuy) and a hip cup system with a circular snap-fit mechanism in combination with two spikes in the inner polar region of the cup (Zimmer Biomet).

This relative motion between cup and PE liner could be a predictor of backside wear. However, features such as the surface structure, specifically roughness of the inner geometry of the acetabular cup, could also have a major impact on backside wear behaviour. Braun et al. showed 45% higher backside wear particles for the Aesculap system (Plasmafit^®^ Plus7) compared to the Zimmer Biomet system (Allofit^®^-S Alloclassic^®^) using an identical experimental setup [9].

In addition, Braun et al. verified that backside wear particles can occur in clinically established cup systems [15]. Furthermore, Reyna et al. was able to detect backside wear caused by micromotion and poor conformity between cup and liner in an in vitro study [25]. As already mentioned, these particles could increase the potential of a resulting inflammatory reaction, which may result in osteolysis. Compared to the in vitro data from Braun et al. and Reyna et al., similar effects could be demonstrated in an in vivo study from Long et al. [26]. In this study, a series of early aseptic loosenings of acetabular cups were identified, an analysis of x-ray images was realized and an optical analysis of the inlays was carried out. The osteolysis that occurred behind the cup could be attributed to backside wear particles in conjunction with optical wear marks on the backside of the PE inlay [26]. Due to the fact that backside wear particles are also significantly smaller than articulating wear particles, they may additionally cause stronger biological responses [9].

However, experimental and clinical studies can show that backside wear in combination with relative motion between the cup and PE liner may have an impact on the osseointegration of the acetabular cup. Furthermore, subsequent studies have to show the characteristics (particle size, roundness and aspect ratio) of the PE particles that may arise and prove their influence on the human body.

From these findings, and the measured results in our study, it could be concluded that there is not a one dimensional relation between a tight fit of the inlay inside the cup, which is reflected in a low relative motion and increased disassembly forces, and the amount of backside wear. In addition, a closer look will be needed at the inner structure of the cup. It is necessary to distinguish between a smooth and a rough surface. A smooth surface with a high rate of relative motion could generate fewer backside wear particles than a rough surface with less motion.

### Limitations

Initially, it should be noted that the fixation of the cups by fixating them in polyurethane does not correspond to the physiological fixation in vivo. However, the advantage of this connection was standardization of the fixation and the focus on the locking mechanisms of the cup systems. Other influencing factors could be excluded. There was a defined impaction force applied on the inlay, but no peak force as it occurs at the impaction by the surgeon.

In the analysis of motion, this work deals with relative motion. The relative motion between cup and inlay, however, could be a combination of plastic and elastic deformation, migration, rotation, creeping and many other factors.

## 5. Conclusions

This experimental study showed significant differences in relative motion at the cup–liner interface and in the disassembly forces for the three investigated locking mechanisms. How relative motion and assembly force affect backside wear in combination with different roughnesses should be analyzed in more detail in a further investigation.

## Figures and Tables

**Table 1:** Mean Relative Motion XYZ ± (µm).

Load Level (kN)	Zimmer Biomet	DePuy	Aesculap
0.5	58.8 ± 32.6	15.2 ± 8.3	6.2 ± 1.1
1.5	74.3 ± 33.7	43.9 ± 14.5	12.5 ± 6.4
2.5	82.8 ± 33.5	66.3 ± 16.1	25.1 ± 8.8
3.5	86.5 ± 32.7	81.1 ± 18.5	38.2 ± 9.9

## References

[1] Crawford R.W., Murray D.W. (1997). Total hip replacement: Indications for surgery and risk factors for failure. Ann. Rheum. Dis..

[2] Pivec R., Johnson A.J., Mears S.C., Mont M.A. (2012). Hip arthroplasty. Lancet.

[3] Makarewich C.A., Anderson M.B., Gililland J.M., Pelt C.E., Peters C.L. (2018). Ten-year survivorship of primary total hip arthroplasty in patients 30 years of age or younger. Bone Jt. J..

[4] Hallab N.J., Jacobs J.J. (2009). Biologic effects of implant debris. Bull. NYU Hosp. Jt. Dis..

[5] Dumbleton J.H., Manley M.T., Edidin A.A. (2002). A literature review of the association between wear rate and osteolysis in total hip arthroplasty. J. Arthroplast..

[6] Harris W.H. (2001). Wear and periprosthetic osteolysis: The problem. Clin. Orthop. Relat. Res..

[7] Prokopovich P. (2014). Interactions between mammalian cells and nano- or micro-sized wear particles: Physico-chemical views against biological approaches. Adv. Colloid Interface Sci..

[8] Akbari A., Roy M.E., Whiteside L.A., Katerberg B.J., Schnettgoecke D.J. (2011). Minimal backside surface changes observed in retrieved acetabular liners. J. Arthroplast..

[9] Braun S., Sonntag R., Schroeder S., Mueller U., Jaeger S., Gotterbarm T., Kretzer J.P. (2019). Backside wear in acetabular hip joint replacement. Acta Biomater..

[10] Kretzer J.P., Zietz C., Schröder C., Reinders J., Middelborg L., Paulus A., Sonntag R., Bader R., Utzschneider S. (2012). Grundlagen zur tribologischen analyse von endoprothesen. Der Orthopäde.

[11] McKellop H.A. (2007). The lexicon of polyethylene wear in artificial joints. Biomaterials.

[12] Kurtz S.M., Ochoa J.A., White C.V., Srivastav S., Cournoyer J. (1998). Backside nonconformity and locking restraints affect liner/shell load transfer mechanisms and relative motion in modular acetabular components for total hip replacement. J. Biomech..

[13] Kyle R.F., Riddle C.D., Kyle M., Rockswold S., Bourgeault C. (2006). Factors influencing the initial micromotion between polyethylene acetabular cups and titanium alloy shells. J. Arthroplast..

[14] Williams V.G., Whiteside L.A., White S.E., McCarthy D.S. (1997). Fixation of ultrahigh-molecular-weight polyethylene liners to metal-backed acetabular cups. J. Arthroplast..

[15] Braun S., Vardag S., Mueller U., Schroeder S., Sonntag R., Bormann T., Gotterbarm T., Kretzer J.P. (2019). Backside wear, particle migration and effectiveness of screw hole plugs in acetabular hip joint replacement with cross-linked polyethylene. Acta Biomater..

[16] Beckmann N.A., Bitsch R.G., Gondan M., Schonhoff M., Jaeger S. (2018). Comparison of the stability of three fixation techniques between porous metal acetabular components and augments. Bone Jt. Res..

[17] ASTM F1820-13 (2013). Standard Test Method for Determining the Forces for Disassembly of Modular Acetabular Devices.

[18] Barrack R.L., Folgueras A., Munn B., Tvetden D., Sharkey P. (1997). Pelvic lysis and polyethylene wear at 5-8 years in an uncemented total hip. Clin. Orthop. Relat. Res..

[19] Bali K., McCalden R.W., Naudie D.D., MacDonald S.J., Teeter M.G. (2016). Backside wear is not dependent on the acetabular socket design in crosslinked polyethylene liners. Clin. Orthop. Relat. Res..

[20] Kligman M., Furman B.D., Padgett D.E., Wright T.M. (2007). Impingement contributes to backside wear and screw-metallic shell fretting in modular acetabular cups. J. Arthroplast..

[21] Lieberman J.R., Kay R.M., Hamlet W.P., Park S.H., Kabo J.M. (1996). Wear of the polyethylene liner-metallic shell interface in modular acetabular components. An in vitro analysis. J. Arthroplast..

[22] Wasielewski R.C., Jacobs J.J., Arthurs B., Rubash H.E. (2005). The acetabular insert-metal backing interface: An additional source of polyethylene wear debris. J. Arthroplast..

[23] Tradonsky S., Postak P.D., Froimson A.I., Greenwald A.S. (1993). A comparison of the disassociation strength of modular acetabular components. Clin. Orthop. Relat. Res..

[24] O’Neill C.K., Napier R.J., Diamond O.J., O’Brien S., Beverland D.E. (2015). Acetabular liner dissociation following total hip arthroplasty: A rare but serious complication that may be easily misinterpreted in the emergency department. Case Rep. Emerg. Med..

[25] Puente Reyna A.L., Jager M., Floerkemeier T., Frecher S., Delank K.S., Schilling C., Grupp T.M. (2016). Backside wear analysis of retrieved acetabular liners with a press-fit locking mechanism in comparison to wear simulation in vitro. BioMed Res. Int..

[26] Long W.J., Nayyar S., Chen K.K., Novikov D., Davidovitch R.I., Vigdorchik J.M. (2018). Early aseptic loosening of the tritanium primary acetabular component with screw fixation. Arthroplast. Today.

